# Higher neutrophil–lymphocyte ratio is associated with depressive symptoms in Japanese general male population

**DOI:** 10.1038/s41598-022-13562-x

**Published:** 2022-06-03

**Authors:** Hirotaka Kinoshita, Daiki Takekawa, Takashi Kudo, Kaori Sawada, Tatsuya Mikami, Kazuyoshi Hirota

**Affiliations:** 1 Department of Anesthesiology, Hirosaki University Graduate School of Medicine, 5 Zaifu-cho, Hirosaki, 036-8562 Japan; 2 Department of Social Medicine, Hirosaki University Graduate School of Medicine, Hirosaki, 036-8562 Japan; 3 Innovation Center for Health Promotion, Hirosaki University Graduate School of Medicine, Hirosaki, 036-8562 Japan

**Keywords:** Depression, Depression

## Abstract

Relationships between the neutrophil–lymphocyte ratio (NLR) and/or the platelet-lymphocyte ratio (PLR) and neuroinflammatory diseases have been reported. Depression is also associated with neuroinflammation. Here, we determined the association between the NLR, PLR, and depressive symptoms. This cross-sectional study is a secondary analysis of the data of the Iwaki Health Promotion Project 2017. We analyzed the characteristics and laboratory data of 1,015 Japanese subjects (597 females, 408 males) including their NLR and PLR values. We assigned the subjects with a Center for Epidemiologic Studies Depression Scale (CES-D) score ≥ 16 to the depressive symptoms group. We performed a multivariate logistic regression analysis to determine whether the NLR and/or PLR were associated with depressive symptoms (CES-D ≥ 16). Two hundred subjects (19.7%; 122 [20.4%] females, 78 [19.1%] males) were assigned to the depressive symptoms group. There were significant differences between the non-depressive symptoms and depressive symptoms groups in the NLR [median (25th to 75th percentile): 1.54 (1.24, 1.97) vs. 1.76 (1.32, 2.37), *P* = 0.005] and the PLR [median (25th to 75th percentile): 123.7 (102.0, 153.9) vs. 136.8 (107.0, 166.5), *P* = 0.047] in males, but not in females. The multivariate logistic regression analysis demonstrated that the NLR was significantly associated with depressive symptoms in males (adjusted odds ratio: per 1 increase, 1.570; 95% confidence interval: 1.120–2.220; *P* = 0.009). In conclusion, our findings indicate that higher NLR may be associated with depressive symptoms in males.

## Introduction

Although the mechanism and pathogenesis of the development of depression are not yet clear, neuroinflammation is known to play an important role in depression^1,2^. The blood concentrations of inflammatory cytokines such as interleukin 6 (IL-6) and tumor necrosis factor-alpha (TNF-α) were reported to increase in patients with depression^3,4^. However, measurement of inflammatory cytokines is expensive and cannot be done in all hospitals.

The neutrophil–lymphocyte ratio (NLR) and the platelet-lymphocyte ratio (PLR), which are calculated as the neutrophil and platelet counts respectively divided by the lymphocyte count, are convenient and inexpensive inflammatory markers. The NLR and the PLR were reported to be positively correlated with IL-6 and TNF-α^5^. Thus, the NLR and the PLR may be novel makers associated with neuroinflammation instead of IL-6 and TNF-α.

We reported that higher preoperative NLR was associated with the development of postoperative delirium that is also caused by neuroinflammation^6,7^. In addition, several studies reported that the NLR and the PLR were associated with generated psychiatric diseases, schizophrenia, attention-deficit/hyperactivity disorder (ADHD), and epileptic seizure^8–10^^.^ Several studies demonstrated that the NLR was significantly higher in patients with depression^11–14^, and a study demonstrated that the PLR was significantly higher in patients with severe depression plus psychotic features compared to patients with mild-to-severe depression^15^. However, four of these five studies had small sample sizes^11–13,15^, and the other study^14^ focused on only patients with diabetes mellitus (DM). Thus, the utility of the NLR and the PLR for the screening of patients with depression has not been elucidated.

The present study was conducted to determine the associations between the NLR and the PLR and depressive symptoms using the data of Japanese community dwellers.

## Methods

### Study design and subjects

This population based cross-sectional study is a secondary analysis of the data of the Iwaki Health Promotion Project 2017 which was approved by the Ethics Committee of the Hirosaki University Graduate School of Medicine (2017–026). Iwaki Health Promotion Project was registered with the University Hospital Medical Information Network (UMIN 000040459, April 1,2014) and this study was conducted in accordance with the recommendations of the Declaration of Helsinki. All participants in this project gave written informed consent for the publication of their data. We herein analyzed the data of the 1,073 Japanese volunteers who live in the Iwaki district of the city of Hirosaki, Japan. Participants with a clinical diagnosis of depression (n = 6) or missing data (n = 52) were excluded from the present analyses, leaving a final total of 1,015 subjects, which means that we conducted a complete case analysis.

### Data collection

Demographic data, medical information, alcohol history and smoking history were obtained from self-reported questionnaires. Regarding to alcohol history, we obtained only current drinking (yes/no), thus we did not assess the amount of alcohol consumption. Blood samples were obtained in the early morning from the medial cubital vein of the subject in a sitting position after he or she had fasted overnight. The following collected blood data were used: peripheral blood cells, aspartate transferase (AST), alanine transferase (ALT), blood urea nitrogen (BUN), creatinine (Cre), hemoglobin A1c (HbA1c), and B-type natriuretic peptide (BNP).

Each subject's NLR and PLR were determined as the absolute neutrophil or platelet count divided by the absolute lymphocyte count, respectively^7^.

### Assessment of depressive symptoms

We used the Center for Epidemiologic Studies Depression Scale (CES-D) to assess the prevalence and severity of depressive symptoms in the subject population. This scale is a short self-report scale designed to measure depressive symptoms in the general population. The maximum score is 60, and higher scores are associated with greater depressive symptoms. Because the reported optimal cutoff CES-D score for the assessment of depressive symptoms in a general population is ≥ 16^16^, we assigned the subjects with CES-D scores ≥ 16 and ≤ 15 to the depressive symptoms and non-depressive symptoms groups, respectively.

### Statistical analyses

Each analysis was performed separately for the males and females, because sex differences in both the NLR and PLR have been reported^13^. All data are presented as the median (25th and 75th percentiles), the number (a percentage of each group), or adjusted odds ratios (aORs) with corresponding 95% confidence intervals (CIs). Differences in continuous variables between two groups were assessed using the Mann–Whitney test, and differences in categorical variables were assessed using Fisher's exact test.

Multivariate logistic regression analyses were performed to examine whether the NLR and/or PLR are associated with depressive symptoms after adjusting for possible confounding factors. The subjects' NLR and PLR values were forced into the model as explanatory variables. As age and body mass index (BMI) were reported to be associated with both the NLR and PLR^17,18^, they were also included to the model. As comorbidities, hypertension, DM, dyslipidemia, coronary artery disease (CAD), and stroke were reported to be associated with depression^19–21^, we included these in the model. In addition, the variables with p-values < 0.2 in univariate analyses were included in the multivariate model not to miss confounders. The variance inflation factor (VIF) was used to check for multicollinearity in each variable. Discrimination was measured by using the area under the curve (AUC). The fitness of the model was evaluated with using Hosmer–Lemeshow test.

All data analyses were performed with EZR software ver. 1.37 (Saitama Medical Center, Jichi Medical University, Saitama, Japan). P-values < 0.05 were considered significant in all tests.

## Results

### The subjects' characteristics

**Figure 1:**
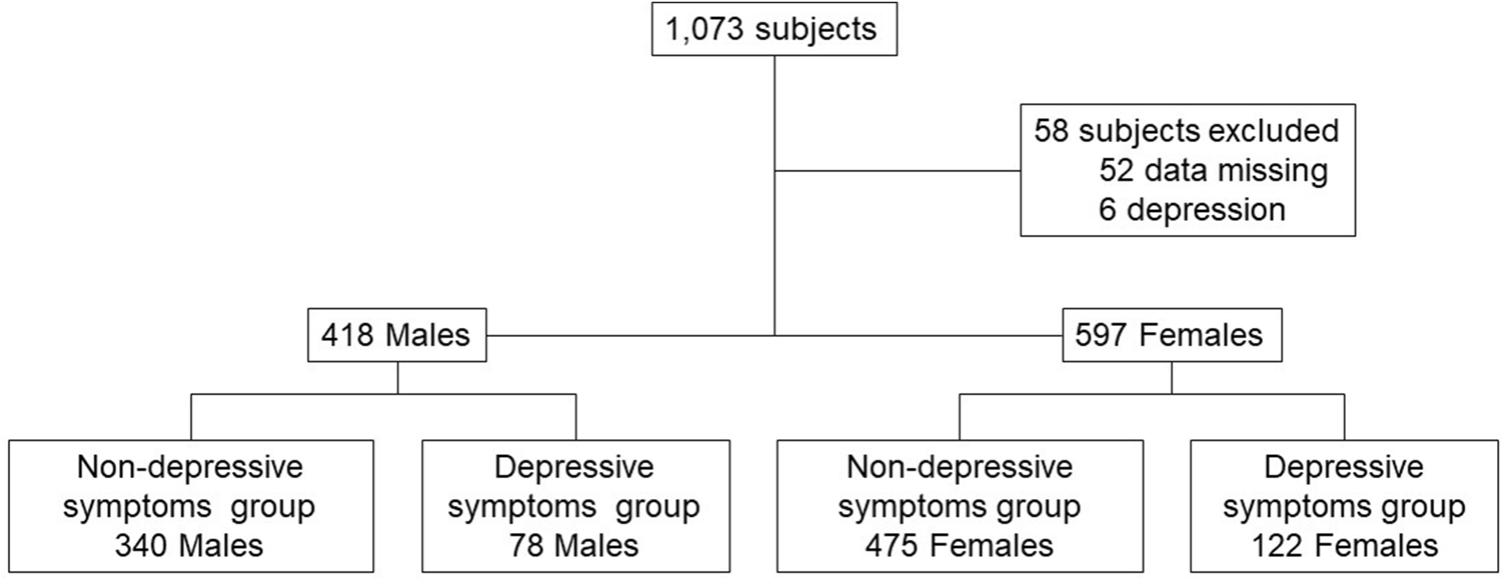
Study population flowchart.

We analyzed the cases of the 1,015 subjects (597 females [59%] and 408 males [41%]) (Fig. 1). The prevalence of depressive symptoms (CES-D score ≥ 16) was 19.7%; we assigned these 200 subjects with depressive symptoms (122 [20.4%] females, 78 [19.1%] males) to the depressive symptoms group.

The subjects' characteristics are summarized in Table 1. There was no significant difference between the depressive symptoms and non-depressive symptoms groups in the subjects' characteristics except for the rate of alcohol drinker in males.

**Table 1:** The subjects' characteristics.

	Males			Females		
	Non-depressive symptoms	Depressive symptoms	*P*-value	Non-depressive symptoms	Depressive symptoms	*P*-value
n	340 (81.3%)	78 (18.7%)	–	475 (79.6%)	122 (20.4%)	–
Age, yrs	55 (41, 67)	50.5 (38, 67)	0.179 0.228	56 (43, 66)	54 (37, 66)	0.179 0.373
< 45	100 (29.4%)	31 (39.7%)		137 (28.8%)	43 (35.2%)	
45 ≤ , < 65	136 (40.0%)	24 (30.8%)		202 (42.5%)	42 (34.4%)	
65 ≤ , < 75	71 (20.9%)	18 (23.1%)		96 (20.2%)	27 (22.1%)	
75 ≤	33 (9.7%)	5 (6.4%)		40 (8.4%)	10 (8.2%)	
Height, cm	169.3 (164.4, 173.2)	168.9 (163.9, 173.7)	0.945	156.4 (151.9, 160.1)	155.5 (151.6, 160.6)	0.796
BW, kg	67.1 (60.9, 73.7)	65.4 (60.5, 73.0)	0.384	52.9 (48.1, 59.1)	53.2 (48.7, 57.5)	0.941
BMI, kg/m^2^	23.5 (21.6, 25.8)	23.4 (21.5, 25.2)	0.422 0.376	22.0 (19.8, 24.2)	22.0 (19.3, 24.7)	0.864 0.375
< 18.5	9 (2.6%)	1 (1.3%)		51 (10.7%)	17 (13.9%)	
18.5 ≤ , < 25	218 (64.1%)	57 (73.1%)		330 (69.5%)	76 (62.3%)	
25 ≤ , < 30	95 (27.9%)	15 (19.2%)		75 (15.8%)	25 (20.5%)	
30 ≤	18 (5.3%)	5 (6.4%)		19 (4.0%)	4 (3.3%)	
CES-D	6 (2, 10)	19 (17, 24.8)	< 0.001*	7 (3, 10)	21 (18, 25)	<0.001*
AST, U/L	23 (19, 29)	23 (19, 27)	0.768	20 (17, 24)	19 (16, 23)	0.070
ALT, U/L	21.5 (17, 31)	22 (18, 30)	0.683	16 (13, 20)	14.5 (11, 19)	0.083
BUN, mg/dL	14.7 (12.5, 17.8)	14.7 (11.9, 17.1)	0.395	13.5 (11.4, 16.1)	13.4 (11.0, 16.8)	0.806
Cre, mg/dL	0.83 (0.75, 0.92)	0.81 (0.74, 0.91)	0.563	0.61 (0.56, 0.67)	0.61 (0.55, 0.67)	0.992
BNP, pg/dL	6.2 (5.8, 11.1)	6.5 (5.8, 10.6)	0.754	9.4 (5.9, 15.1)	10.0 (6.0, 16.3)	0.832
HbA1c, %	5.6 (5.4, 5.8)	5.6 (5.3, 5.9)	0.353	5.8 (5.4, 5.8)	5.6 (5.3, 5.8)	0.578
Hypertension	106 (31.2%)	21 (26.9%)	0.498	115 (24.2%)	34 (27.9%)	0.413
DM	25 (7.4%)	6 (7.7%)	1.000	17 (3.6%)	3 (2.5%)	0.778
Dyslipidemia	42 (12.4%)	9 (11.5%)	1.000	56 (11.8%)	17 (13.9%)	0.536
CAD	8 (2.4%)	0 (0%)	0.361	7 (1.5%)	1 (0.8%)	1.000
Stroke	9 (2.6%)	1 (1.3%)	0.696	8 (1.7%)	3 (2.5%)	0.704
Current smoker	89 (26.2%)	19 (24.4%)	0.777	34 (7.2%)	13 (10.7%)	0.257
Alcohol drinker	245 (72.1%)	45 (57.7%)	0.020*	156 (32.8%)	37 (30.3%)	0.665

*P < 0.05. Differences between the non-depressive symptoms and depressive symptoms groups were examined by Fisher's exact test for categorical variables and Mann–Whitney test for continuous variables. Data are shown as number (a percentage of each group) or median (25 to 75th percentile).

*ALT* alanine transferase, *AST* aspartate transferase, *BMI* body mass index, *BNP* B-type natriuretic peptide, *BUN* blood urea nitrogen, *BW* body weight, *CAD* coronary artery disease, *CES-D* Center for Epidemiologic Studies Depression Scale, *Cre* creatinine, *DM* diabetes mellitus, *HbA1c* hemoglobin A1c.

### The NLR, the PLR, and depression

There were significant differences between two groups in the NLR [median (25th to 75th percentile): 1.54 (1.24, 1.97) vs. 1.76 (1.32, 2.37), *P* = 0.005] and the PLR [median (25th to 75th percentile): 123.7 (102.0, 153.9) vs. 136.8 (107.0, 166.5), *P* = 0.047] in males, but not in females (Table 2). The multivariate logistic regression analysis demonstrated that the NLR was significantly associated with depressive symptoms in males (aOR: per 1 increase, 1.570; 95% confidence interval: 1.120–2.220; *P* = 0.009) (Table 3). The multivariate logistic regression analysis also showed that alcohol drinker was significantly associated with depressive symptoms in males (aOR:0.548; 95% CI: 0.322–0.930; *P* = 0.026) (Table 3).

**Table 2:** Relationships between depressive symptoms and NLR or PLR.

	Males			Females		
	Non-depressive symptoms	Depressive symptoms	*P*-value	Non-depressive symptoms	Depressive symptoms	*P*-value
NLR	1.54 (1.24, 1.97)	1.76 (1.32, 2.37)	0.005*	1.49 (1.16, 1.97)	1.56 (1.23, 2.03)	0.373
PLR	123.7 (102.0, 153.9)	136.8 (107.0, 166.5)	0.047*	139.4 (113.2, 173.0)	135.0 (114.4, 165.8)	0.801

*P < 0.05. Differences between the non-depressive symptoms and depressive symptoms groups were estimated by the Mann–Whitney test. Data are median (25th to 75th percentile), NLR: neutrophil–lymphocyte ratio, PLR: platelet-lymphocyte ratio.

**Table 3:** Logistic regression analysis to identify whether NLR and PLR are associated with depressive symptoms.

	Males		Females	
	aOR (95%CI)	*P*-value	aOR (95%CI)	*P*-value
NLR, per 1 increase	1.570 (1.120–2.220)	0.009*	0.871 (0.652–1.160)	0.349
PLR, per 1 increase	1.000 (0.994–1.010)	0.899	1.000 (0.997–10.10)	0.520
**Age**				
< 45	Reference		Reference	
45 ≤ , < 65	0.546 (0.285, 1.050)	0.069	0.668 (0.392, 1.140)	0.137
65 ≤ , < 75	0.763 (0.344, 1.690)	0.504	0.912 (0.463, 1.800)	0.790
75 ≤	0.359 (0.111, 1.160)	0.087	0.734 (0.289, 1.860)	0.516
**BMI**				
18.5 ≤ , < 25	Reference		Reference	
< 18.5	0.355 (0.043, 2.920)	0.335	1.470 (0.780, 2.790)	0.233
25 ≤ , < 30	0.674 (0.352, 1.290)	0.235	1.400 (0.812, 2.420)	0.225
30 ≤	0.837 (0.279, 2.510)	0.751	0.822 (0.258, 2.620)	0.741
AST, per 1 U/L increase	–		0.951 (0.900–1.010)	0.077
ALT, per 1 U/L increase	–		1.020 (0.986–1.050)	0.247
Hypertension	1.060 (0.523–2.130)	0.881	1.340 (0.766–2.340)	0.305
DM	1.030 (0.355–2.960)	0.964	0.565 (0.151–2.110)	0.397
Dyslipidemia	1.080 (0.446–2.600)	0.871	1.360 (0.706–2.630)	0.357
CAD	–		0.476 (0.056–4.040)	0.496
Stroke	1.000 (1.000–1.000)	0.551	1.000 (1.000–1.000)	0.596
Alcohol drinker	0.548 (0.322–0.930)	0.026*		

*P < 0.05. As none of the VIF values were up to 10, there was no collinearity in the model. AUC values for males and females were 0.675 and 0.603, respectively. Hosmer–Lemeshow test: *P* = 0.36, which means that the fitness of this model was good.

*aOR* adjusted odds ratio, *ALT* alanine transferase, *AST* aspartate transferase, *BMI* body mass index, *CAD* coronary artery disease, *DM* diabetes mellitus, *NLR* neutrophil–lymphocyte ratio, *PLR* platelet-lymphocyte ratio.

## Discussion

We examined the cross-sectional association between the NLR and the PLR and depressive symptoms in a general population. The results of our analyses demonstrated that after the adjustment for possible confounding factors, higher NLR was significantly associated with depressive symptoms in males. In addition, alcohol drinker was significantly associated with a lower risk of depressive symptoms in males.

Neuroinflammation caused by a chronic activation of microglia (which produce excessive levels of inflammatory cytokines) is involved in the mechanism of depression^22^. Both the NLR and the PLR are known to be possible inflammatory markers^6–10^. In the present study, although the univariate analysis indicated that both the NLR and the PLR were significantly higher in the depressive symptoms group than in the non- depressive symptoms group, the multivariate analysis revealed that only higher NLR was associated with the development of depressive symptoms in only males after adjusting for possible confounders. In contrast, Kayhan et al. reported that the PLR was significantly increased according to the severity of depression. The difference between our findings and their data may be due to the study population^15^. Their study didn’t include healthy controls, whereas our study included healthy subjects without clinically diagnosed depression.

The lifespans of neutrophils and platelets in human are 24 h^23^ and 10 days^24^, respectively. Indeed, our previous study showed that postoperative NLR significantly increased from preoperative NLR, but the PLR wasn’t changed before and after surgery^7^. On the other hand, patients with a clinical diagnosis depression are reported to develop a chronic low-grade inflammation^25^, and the PLR is reported to increase according to the severity of depression^15^. The NLR and PLR may thus reflect acute and chronic inflammation, respectively.

The NLR may also promptly reflect the effects of treatment, as a previous study showed that the improvement in depressive symptoms provided by treatment with selective serotonin inhibitors was accompanied by a reduction in the patients' NLR^12^.

To the best of our knowledge, the present study is the first to demonstrate the association between higher NLR and depressive symptoms in males but not in females. Although the mechanisms underlying sex-related differences in the NLR remain unclear, the estrogen level may be attributed to the difference^26,27^. Wu et al. reported that the NLR at the ages of 30–49 years was higher in females than in males, while the NLR at the ages of 60–69 was higher in males than in females^17^. This may be due to postmenopausal estrogen depletion. Indeed, we observed a significant but weak negative correlation between age and the NLR in females (Β =  − 0.005, R^2^ = 0.007, *P* = 0.037). A large cohort study is needed to determine whether the NLR can be used as a potential reference for the development of depressive symptoms.

We also observed that alcohol drinker was significantly associated with a lower risk of depressive symptoms in males. Similarly, Gémes et al. reported that light-to-moderate alcohol consumption was associated with the lowest risk of depression^28^. On the other hand, it was also reported that excessive alcohol drinking was associated with development of depression^29^. Thus, the amount of alcohol consumption may be associated with the development of depression. However, as we did not assess the amount of alcohol consumption in the present study, this result should be treated with caution.

There are some limitations of our study. First, as this was a retrospective cross-sectional study, we cannot provide definite information about cause-and-effect relationships and changes in the NLR and depressive symptoms over time. Additionally, there might be undetected confounding factors that affected the results. Second, as this study was limited to Japanese subjects living in a specific area, possible racial and regional differences were not considered. Third, as we did not include patients with a clinical diagnosis of depression to rule out the effects of antidepressants on depressive symptoms and neuroinflammation, our results might not be applicable to all patients with depression. Patients with a clinical diagnosis of depression are often treated with antidepressants. Some antidepressants can modulate microglial activation that plays a key role in neuroinflammation^30^. Thus, in the patients with a clinical diagnosis of depression, neuroinflammation may not necessarily reflected the CES-D scores. On the other hand, this result suggests the potential of the NLR for screening depressive symptoms in general populations especially in males, since our logistic regression analysis showed that higher NLR was significantly associated with depressive symptoms in males. Fourth, as we collected medical history by self-reported questionnaires, there was a possibility that patients with depression who didn’t report illness or who didn’t seek medical care were included to the present study.

In conclusion, this population based cross-sectional study demonstrated that higher NLR was significantly associated with depressive symptoms in males but not in females. Prospective longitudinal studies are required to confirm the relationships between the NLR and depressive symptoms.

## Data Availability

The datasets used and analyzed in the current study are available from the corresponding author on reasonable request.
