# Publisher Correction: Higher neutrophil–lymphocyte ratio is associated with depressive symptoms in Japanese general male population

**DOI:** 10.1038/s41598-022-15967-0

**Published:** 2022-07-11

**Authors:** Hirotaka Kinoshita, Daiki Takekawa, Takashi Kudo, Kaori Sawada, Tatsuya Mikami, Kazuyoshi Hirota

**Affiliations:** 1 Department of Anesthesiology, Hirosaki University Graduate School of Medicine, 5 Zaifu-cho, Hirosaki, 036-8562 Japan; 2 Department of Social Medicine, Hirosaki University Graduate School of Medicine, Hirosaki, 036-8562 Japan; 3 Innovation Center for Health Promotion, Hirosaki University Graduate School of Medicine, Hirosaki, 036-8562 Japan

Correction to: *Scientific Reports* 10.1038/s41598-022-13562-x, published online 03 June 2022

The original version of this Article contained errors in Table 1, where the column ‘*P*-value’ was incomplete for ‘Females’. Furthermore, data in the row ‘CES-D’ contained errors, where the *p*-value for ‘Males’ and the data ‘Non-depressive symptoms’ for ‘Females’ was displaced, and data for ‘Depressive symptoms’ for ‘Females’ was omitted.

The original Table 1 and accompanying legend appear below.

**Table 1:** The subjects' characteristics.

	Males			Females		
	Non-depressive symptoms	Depressive symptoms	*P*-value	Non-depressive symptoms	Depressive symptoms	*P*-value
n	340 (81.3%)	78 (18.7%)	–	475 (79.6%)	122 (20.4%)	–
Age, yrs	55 (41, 67)	50.5 (38, 67)	0.179 0.228	56 (43, 66)	54 (37, 66)	0.179 0.373
< 45	100 (29.4%)	31 (39.7%)		137 (28.8%)	43 (35.2%)	
45 ≤ , < 65	136 (40.0%)	24 (30.8%)		202 (42.5%)	42 (34.4%)	
65 ≤ , < 75	71 (20.9%)	18 (23.1%)		96 (20.2%)	27 (22.1%)	
75 ≤	33 (9.7%)	5 (6.4%)		40 (8.4%)	10 (8.2%)	
Height, cm	169.3 (164.4, 173.2)	168.9 (163.9, 173.7)	0.945	156.4 (151.9, 160.1)	155.5 (151.6, 160.6)	0.796
BW, kg	67.1 (60.9, 73.7)	65.4 (60.5, 73.0)	0.384	52.9 (48.1, 59.1)	53.2 (48.7, 57.5)	0.941
BMI, kg/m^2^	23.5 (21.6, 25.8)	23.4 (21.5, 25.2)	0.422 0.376	22.0 (19.8, 24.2)	22.0 (19.3, 24.7)	0.864 0.375
< 18.5	9 (2.6%)	1 (1.3%)		51 (10.7%)	17 (13.9%)	
18.5 ≤ , < 25	218 (64.1%)	57 (73.1%)		330 (69.5%)	76 (62.3%)	
25 ≤ , < 30	95 (27.9%)	15 (19.2%)		75 (15.8%)	25 (20.5%)	
30 ≤	18 (5.3%)	5 (6.4%)		19 (4.0%)	4 (3.3%)	
CES-D	6 (2, 10)	19 (17, 24.8)		< 0.001*	7 (3, 10)	
AST, U/L	23 (19, 29)	23 (19, 27)	0.768	20 (17, 24)	19 (16, 23)	
ALT, U/L	21.5 (17, 31)	22 (18, 30)	0.683	16 (13, 20)	14.5 (11, 19)	
BUN, mg/dL	14.7 (12.5, 17.8)	14.7 (11.9, 17.1)	0.395	13.5 (11.4, 16.1)	13.4 (11.0, 16.8)	
Cre, mg/dL	0.83 (0.75, 0.92)	0.81 (0.74, 0.91)	0.563	0.61 (0.56, 0.67)	0.61 (0.55, 0.67)	
BNP, pg/dL	6.2 (5.8, 11.1)	6.5 (5.8, 10.6)	0.754	9.4 (5.9, 15.1)	10.0 (6.0, 16.3)	
HbA1c, %	5.6 (5.4, 5.8)	5.6 (5.3, 5.9)	0.353	5.8 (5.4, 5.8)	5.6 (5.3, 5.8)	
Hypertension	106 (31.2%)	21 (26.9%)	0.498	115 (24.2%)	34 (27.9%)	
DM	25 (7.4%)	6 (7.7%)	1.000	17 (3.6%)	3 (2.5%)	
Dyslipidemia	42 (12.4%)	9 (11.5%)	1.000	56 (11.8%)	17 (13.9%)	
CAD	8 (2.4%)	0 (0%)	0.361	7 (1.5%)	1 (0.8%)	
Stroke	9 (2.6%)	1 (1.3%)	0.696	8 (1.7%)	3 (2.5%)	
Current smoker	89 (26.2%)	19 (24.4%)	0.777	34 (7.2%)	13 (10.7%)	
Alcohol drinker	245 (72.1%)	45 (57.7%)	0.020*	156 (32.8%)	37 (30.3%)	

*P < 0.05. Differences between the non-depressive symptoms and depressive symptoms groups were examined by Fisher's exact test for categorical variables and Mann–Whitney test for continuous variables. Data are shown as number (a percentage of each group) or median (25 to 75th percentile).

*ALT* alanine transferase, *AST* aspartate transferase, *BMI* body mass index, *BNP* B-type natriuretic peptide, *BUN* blood urea nitrogen, *BW* body weight, *CAD* coronary artery disease, *CES-D* Center for Epidemiologic Studies Depression Scale, *Cre* creatinine, *DM* diabetes mellitus, *HbA1c* hemoglobin A1c.

The original Article has been corrected.

